# What proteomics has taught us about honey bee (*Apis mellifera*) health and disease

**DOI:** 10.1002/pmic.202400075

**Published:** 2024-06-19

**Authors:** Maor Arad, Kenneth Ku, Connor Frey, Rhien Hare, Alison McAfee, Golfam Ghafourifar, Leonard J. Foster

**Affiliations:** ^1^ Department of Chemistry University of the Fraser Valley Abbotsford BC Canada; ^2^ Department of Biochemistry and Molecular Biology Michael Smith Laboratories University of British Columbia Vancouver BC Canada; ^3^ Department of Medicine University of British Columbia Vancouver BC Canada; ^4^ Faculty of Health Sciences Simon Fraser University Burnaby BC Canada; ^5^ Department of Applied Ecology North Carolina State University Raleigh North Carolina USA

**Keywords:** biomarkers, climate change, health monitoring, honey bees (*Apis mellifera*), proteomics

## Abstract

The Western honey bee, *Apis mellifera*, is currently navigating a gauntlet of environmental pressures, including the persistent threat of parasites, pathogens, and climate change – all of which compromise the vitality of honey bee colonies. The repercussions of their declining health extend beyond the immediate concerns of apiarists, potentially imposing economic burdens on society through diminished agricultural productivity. Hence, there is an imperative to devise innovative monitoring techniques for assessing the health of honey bee populations. Proteomics, recognized for its proficiency in biomarker identification and protein–protein interactions, is poised to play a pivotal role in this regard. It offers a promising avenue for monitoring and enhancing the resilience of honey bee colonies, thereby contributing to the stability of global food supplies. This review delves into the recent proteomic studies of *A. mellifera*, highlighting specific proteins of interest and envisioning the potential of proteomics to improve sustainable beekeeping practices amidst the challenges of a changing planet.

**Abbreviations**
ABPV: acute bee paralysis virus
AFB: American foulbrood
AMP: antimicrobial peptides
BQCV: black queen cell virus
CBPV: chronic bee paralysis virus
DWV: deformed wing virus
IAPV: Israeli acute paralysis virus
KBV: Kashmir bee virus
SBV: sacbrood virus
VSH: *Varroa*‐sensitive hygiene

## 1 INTRODUCTION

**FIGURE 1:**
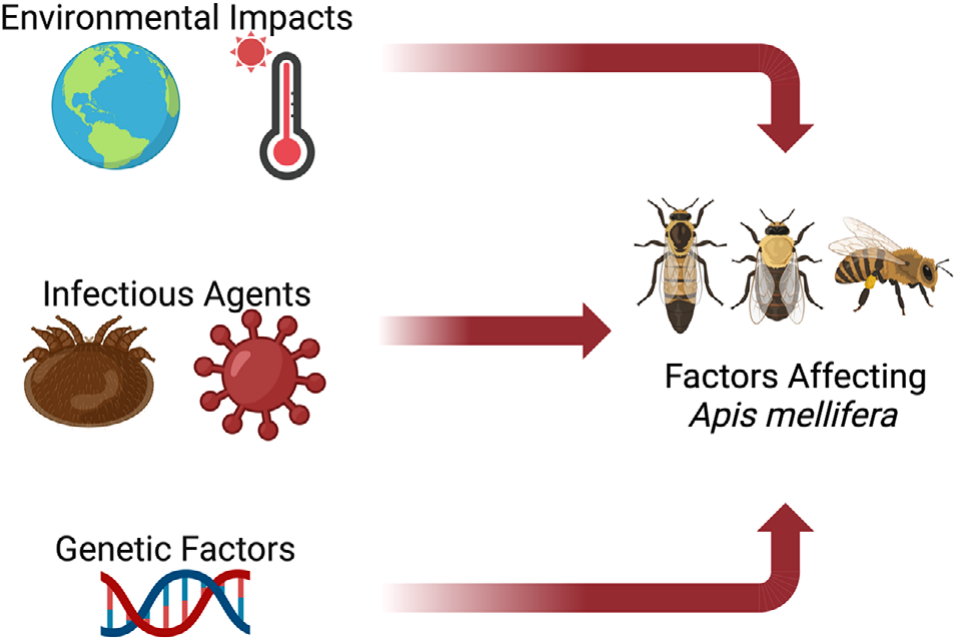
Factors affecting the health of *Apis mellifera*. An *A. mellifera* colony is comprised of a queen, seasonally produced drones, and workers (left to right), each with specialized roles and physiological features. Most research has focused on workers, but our understanding of how each caste and sex is affected by different stressors is growing and is one topic to which proteomics can contribute an improved understanding. Figure created with BioRender.com.

Environmental impacts, infectious agents, and genetic factors have profound impacts on the diversity of life on Earth, even affecting the highly adaptable honey bee (*Apis mellifera*) (Figure 1). Honey bees are crucial for crop pollination, and the domestication and longevity of their colonies make them a useful indicator for environmental conditions [1, 2]. Notably, recent winter colony losses have been substantial in North America, with winter losses ranging from 23.2% to 45.5% in Canada and 20.2% to 37.4% in the USA over the last 5 years (2018–2023) [3, 4]. Impactful winter losses have been reported throughout European countries as well, with an overall average loss of 17.7% (2019–2020), with localized losses reaching as high as 36.5% in Spain [5]. This has led to costly requirements of beekeepers to supplement their losses by splitting their healthy colonies into new nucleus colonies (nucs) to maintain their operations.

Omics‐based research methods (genomics, transcriptomics, metabolomics, etc.) facilitate the study of *A. mellifera*, with proteomics being particularly promising for health monitoring. Metabolomics is a developing field and offers valuable insights into small‐molecule profiles, but still faces analytical challenges in detection and quantification [6]. Although genomics is normally the preferred method for identifying biomarkers, the extreme genetic recombination rate in honey bees [7] means that candidate mutations (markers), which do differ between castes and developmental stages, must be causal, or their linkages to traits quickly decay. This is problematic because causality is difficult to validate without performing gene editing experiments, and some traits may require entire colonies to possess the mutation for the trait to manifest; a feat which has not yet been performed. Moreover, genetic markers are normally static with respect to their environment, whereas the expressed genome responds to stimuli within the organism's lifetime and is thus a reflection of current, rather than evolutionary, conditions.

While both proteomics and transcriptomics assess the expressed genome, proteomics quantifies proteins directly; therefore, the data better represent the state of cellular functions than mRNA transcripts, which are one step removed from being a functional agent and poorly correlate with protein abundance [8, 9, 10, 11, 12, 13]. Proteins often also function as part of protein complexes – with network disruption conceivably being influenced by pathogens and abiotic stressors – and proteomics is the only technique available that can identify and evaluate these complexes in a high‐throughput way [14], which has recently been applied to honey bees [15]. From a practical perspective, proteins are also more stable than transcripts and are less prone to degradation during transport from field sites to the laboratory [16].

In this review, we discuss proteomics as a tool for honey bee health monitoring. We envision that proteomics could be used to identify, quantify, and track panels of protein indicators for different traits and health status in honey bee populations. Proteomics has already identified key proteins that reflect honey bee adaptation to environmental changes, and, in a first‐of‐its kind endeavor, proteomic studies have even produced demonstrably effective biomarkers for selective breeding [17]. Beyond biomarker discovery, the advancement of proteomic methods can further enable using honey bees as a model for studying environmental impacts [18].

## 2 PROTEOMIC ANALYSIS

**FIGURE 2:**
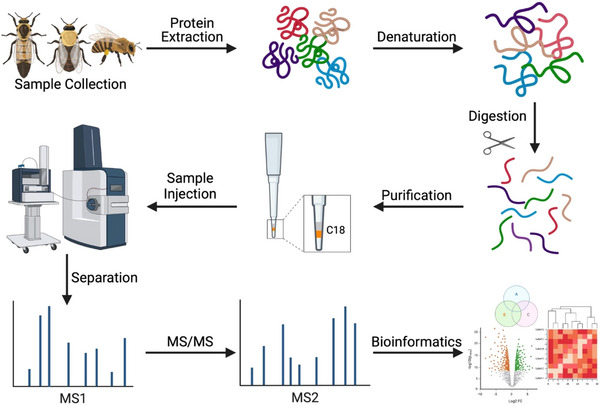
Overview of the bottom‐up proteomic analysis workflow. Steps for processing samples from *A. mellifera* follow the same general procedure as for any tissue, except the protein extraction step may require intense mechanical disruption (e.g., high‐frequency ceramic bead beating) to break apart the tough exoskeleton. Figure created with BioRender.com.

Proteomic studies can employ two main workflows: top‐down and bottom‐up. The top‐down approach involves direct analysis of proteins via mass spectrometry (MS) but is less commonly used due to incompatibility with large proteins and low sensitivity, among other challenges [19, 20, 21]. Bottom‐up proteomics (Figure 2) breaks down proteins into peptides using enzymes such as trypsin (the most common enzyme for this purpose) for easier MS analysis, despite some sample losses and computational challenges [reviewed in 22]. However, like all enzymes, using trypsin comes with certain limitations, such as possible missed cleavages and the “dark proteome” (protein segments that do not yield peptides compatible with standard MS data acquisition methods) that can affect the downstream analysis [23].

While polymerase chain reaction (PCR)‐based methods for disease detection and gene expression analysis are unrivalled in their speed, low cost, and sensitivity, one advantage of using proteomics for health monitoring is that thousands of proteins are analyzed at once. This means that, although PCR is the most sensitive method for pathogen detection, proteomic data provides insight into whether the pathogen is having a measurable impact on host physiology. In addition, while analyzing a proteomic sample is costly, the cost‐per‐test ratio can be reduced by evaluating multiple targets, such as specific pathogen proteins as well as general markers of health, toxicity, or disease, in a single sample. Moreover, the samples themselves can be multiplexed using isotopic or isobaric labeling strategies (e.g., dimethyl labeling or tandem mass tags) to further reduce the cost by a factor of three to five. Using these strategies, the real cost‐per‐test ratio is relatively low, and the challenge will lie in identifying robust markers for the health status of interest which do not covary with extraneous variables.

## 3 SEASONAL AND TEMPERATURE EFFECTS ON HONEY BEE PHYSIOLOGY

The seasonal dynamics of *A. mellifera* populations and behaviors are critically influenced by their adaptive strategies for survival and colony expansion, as they transition from the resource‐scarce winter to the productive summer months [24, 25, 26]. During the summer, the adult lifespan for worker bees is typically 15–38 days, which is much shorter than the shoulder season (30–60 days) and winter bee lifespan (∼140 days) [27]. From spring to late summer, the workers’ main activities are centered on nursing the brood and foraging for resources [26]. When temperatures and food availability rise in late winter and early spring, the queen bee intensifies the production of offspring, replenishing the sparse winter population of 10,000–15,000 bees with the larger summer colonies, in which bees can number up to 50,000 [26].

Numerous studies have demonstrated that *A. mellifera* exhibits seasonal variations in protein expression [28, 29, 30, 31]. A noteworthy study by Ward et al. [31] utilized 2D gel electrophoresis, quantitative (q)PCR, and label‐free quantitative MS to analyze the protein profiles in the head, abdomen, and venom sacs of *A. mellifera* from summer and winter populations. They discovered numerous proteins in these tissues that were exclusively present during the summer, and were undetectable, falling below the sensitivity threshold, in winter bees [31]. The winter bees had a restricted proteome across all three anatomical sites, which may somehow be linked to their increased longevity. The winter samples showed increased expression of xenobiotic metabolism proteins, vitellogenin proteins, and antioxidants [31]. These elevated levels of antioxidant proteins in the winter bees suggest a specialized metabolic system that reduces cellular oxidative stress, enhancing winter bee longevity [31, 32]. Future studies that consider collecting samples from additional body parts, like the fat body and brain, could give a more comprehensive understanding of seasonal physiological changes. Studying these changes in queen bees could also be useful, as the winter is one of the few times when egg production naturally ceases [33], which comes with many physiological changes which are yet to be investigated.

Vitellogenin, a multifunctional yolk protein, is associated with immunity and lifespan in honey bees, making it a potential biomarker for assessing their health [34, 35, 36, 37]. With an important role in oogenesis and antioxidant activity, vitellogenin is expressed at elevated levels in winter bees and is the dominant protein in the hemolymph of winter worker bees [25, 38]. Interestingly, this substance also acts as a carrier for a variety of macromolecules, including carbohydrates, lipids, metal ions, vitamins, and hormones [39, 40]. Many studies have used reverse transcription (RT)‐PCR, qPCR, and proteomic analysis to investigate the protein's diverse functions, and it is a good candidate to assess as a biomarker for honey bee health monitoring [31, 36, 37, 41–43].

Research on how changes in temperature affect honey bees has highlighted, unsurprisingly, the crucial role of heat shock proteins (HSPs) as primary mechanisms to mitigate the strain of extreme heat events [44, 45, 46, 47]. Studying how heat affects reproduction has yielded valuable information on how extreme heat events can reduce fertility [48, 49], and future research should aim to determine whether selective breeding or stock enrichment could be employed to counteract negative effects. Research aimed at pinpointing protein markers for heat stress, as a signal of adverse shipping conditions, has been unsuccessful because candidate biomarkers are influenced by both heat‐shock and viral infections, complicating their diagnostic utility [47, 50, 51]. This highlights the complexity of using expression biomarkers to monitor bee health. Protein levels that respond to environmental factors offer diagnostic potential, yet their interpretation becomes challenging when a single protein is influenced by different stressors, which might also cloud the diagnostic picture or lead to incorrect conclusions. However, such challenges may be overcome by identifying and measuring the covariates needed to deconvolute multifunctional marker interpretation (in this case, by quantifying viral infections).

## 4 IMMUNITY AND SPERM VIABILITY

The reproduction‐immunity trade‐off illustrates a crucial conundrum: While both sexual potency and a robust immune system are assets for species longevity, they may inversely affect each other [52, 53] in what can be boiled down to a compromise due to resource limitation. Interestingly, this trade‐off has been demonstrated in some female insects that store sperm, as queen honey bees do [54, 55, 56, 57]. In queen honey bees, evolutionary forces have favored the reduction of cytotoxic immune effectors, such as reactive nitrogen species (RNS) and reactive oxygen species (ROS), to help preserve sperm over an extended period. This is complemented by the upregulation of enzymes that mitigate oxidative stress (including vitellogenin), indicating a potential trade‐off between immunity and sperm longevity. However, further research is needed to determine if this causes a bona fide immunity handicap in honey bees. While proteomics analysis has revealed some evidence supporting a reproduction‐immunity trade‐off in queen honey bees [47, 58], drone honey bees are heavily invested in reproduction but appear not to have reduced immunocompetence [59], and evidence in other species is mixed or contrary [53]. By applying proteomics to analyze immune protein expression, sperm viability, and resource allocation trade‐offs, we move toward establishing a better understanding of the hierarchy of factors influencing reproductive health, which will enable us to better direct resources for preserving their vitality.

Proteomics is an excellent tool for investigating tissues like the queen's spermathecal fluid, which is a proteinaceous secretion where transcription does not occur, thus facilitating the study of proteins directly involved in sperm maintenance. Five spermathecal fluid proteins significantly correlating with sperm viability were identified by McAfee et al. [47] odorant binding protein 14 (OBP14), lysozyme, serpin 88Ea, artichoke, and heat shock protein 10 (HSP10). While interesting, the relationships are correlational and without more knowledge of specific protein functions, our ability to interpret the data is limited. Conducting more mechanistic studies, using techniques such as RNAi or CRISPR to characterize gene functions, would be invaluable for interpreting proteomic and other high‐throughput data. The current deficiency in functional knowledge restricts our ability to capitalize on systems biology techniques and underscores the importance of enhancing our understanding of protein functions in honey bees.

## 5 THE ROLE OF PROTEOMICS IN HEALTH MONITORING

Bees face many diseases, like bacterial, fungal, parasitic, and viral infections, which affect them in different ways and require us to understand their biology to keep hives healthy. Numerous methods can be employed to study bee diseases; however, proteomics offers an exceptional avenue for analyzing protein–protein interactions, identification of biomarkers, and enhancement of disease detection. Importantly, protein–protein interactions and their disruptions can control healthy and diseased states in other organisms [60], and while we expect the same to be true in honey bees, this topic has not been thoroughly researched. The only study yet investigating the impact of a pathogen on protein complexation is a preliminary investigation into effects of *Vairimorpha* spp. (formerly *Nosema*) [61] infection on protein–protein interaction profiles within the honey bee midgut [62]. With this demonstration, we hope that more studies will begin to map the honey bee interactome and how it is perturbed in disease states.

### 5.1 Bacterial diseases

#### 5.1.1 American foulbrood

**FIGURE 3:**
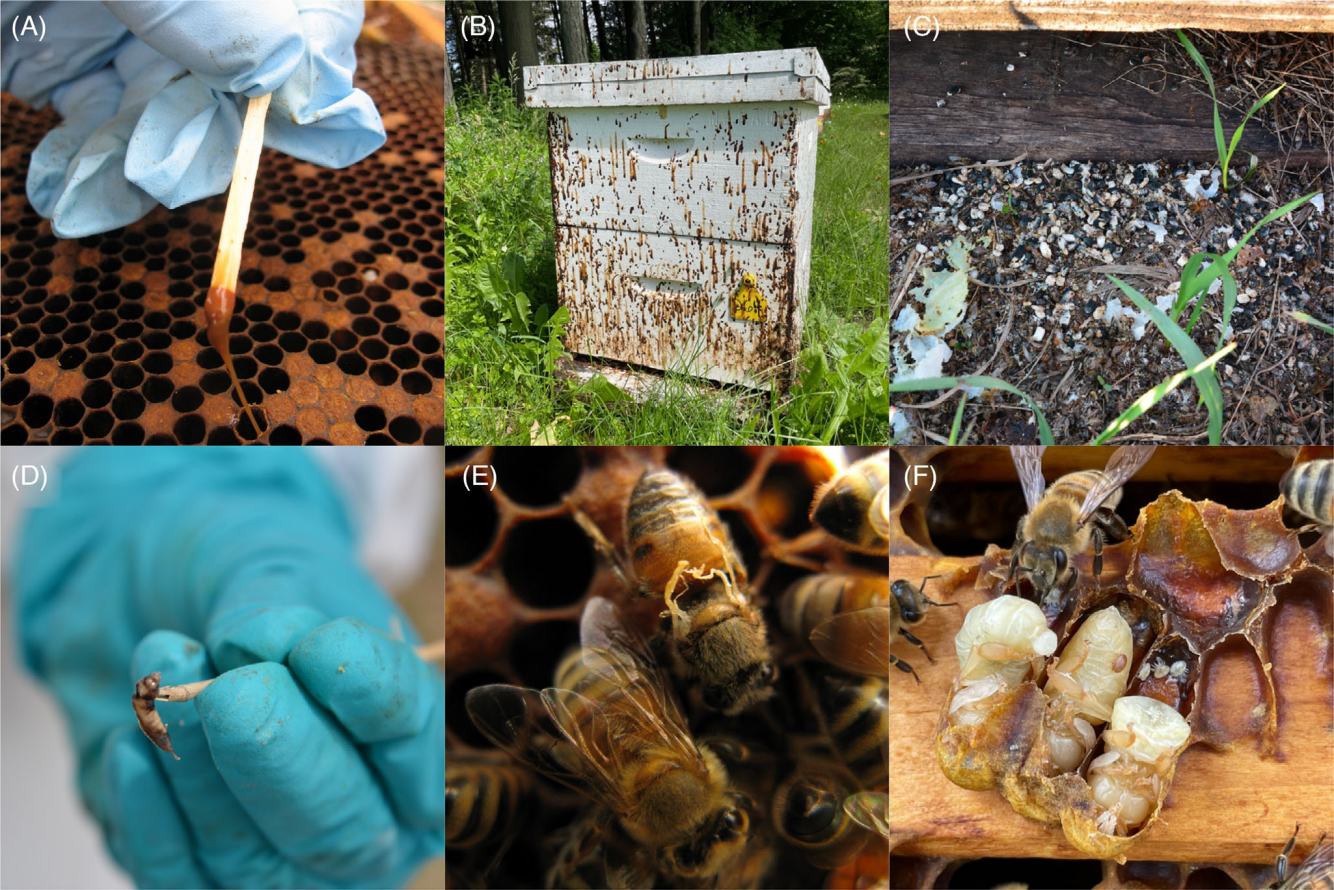
Visual presentation of various diseases affecting *Apis mellifera*. (A) American foulbrood. (B) *Vairimorpha apis*. (C) Chalkbrood. (D) Sacbrood. (E) Deformed wing virus. (F) *Varroa* mites. Images provided by Paul Kozak – Ontario Ministry of Agriculture, Food and Rural Affairs (A, E), Laurence Plamondon (B, F), and Shelly Hoover (C, D).

American foulbrood (AFB), a disease targeting honey bee larvae, presents variable clinical signs that hinge on the genotype of the infecting *Paenibacillus larvae* (the pathogenic agent), and to a lesser degree the vitality and inherent resistance of the bee colony [63, 64]. Shortly after brood cells are capped, abnormalities in the larvae's color, structure, and consistency arise, characterized by irregular capping patterns that stand out among healthy brood cells (Figure 3A). Larvae presenting with AFB experience mortality within a period of 3−12 days after infection, sometimes even before the cells of the brood are sealed [65, 66, 67].

There have been a few proteomic studies on *P. larvae* that investigated their virulence factors [68, 69]. Fünfhaus and Genersch first detected the presence of a S‐layer protein in one of the four *P. larvae* genotype classifications, ERIC II [69]. A later study by Erban et al. focused on the quantitative comparison of previously and newly identified protein‐based virulence factors [68]. They noted different proteins identified between the four classifications, which correlated with their respective effects on honey bee larvae mortality [68].

Erban et al. also utilized proteomic techniques to detect bacterial virulence factors in the colony, identifying six proteins – SpIA, PICBP49, enolase, DnaK, S‐layer protein, and a bacteriocin – that were consistent with markers of infection documented in prior studies [65, 70–74]. Chan et al. applied MS techniques to examine honey bees’ response to *P. larvae*, revealing immune responses of 5‐day old larvae [75]. These included variations in antimicrobial peptides (AMPs), phenoloxidase enzymes, and pathogen recognition proteins. Notably, the phenoloxidase enzyme was of special interest because of its association with resistance to *P. larvae* and its elevated expression levels during infection [75]. Kim et al. found that *P. larvae*‐exposed bees had differential expression of major royal jelly proteins 1–7, AMPs, and vitellogenin, providing possible biomarkers for the disease [76]. However, as these proteins are also affected by other factors (such as other pathogens and age) [36, 76–78], their expression is not indicative of this specific condition. Proteins such as these may be better thought of as general health indicators, as opposed to a diagnostic tool for a specific disease. Such broad biomarkers hold potential for improving overall health surveillance, whether to address existing infections or as a preventive intervention.

Although challenging, biomarkers indicating a specific trait or condition can indeed be developed. A pivotal study by Guarna et al. showcased the ability of selective breeding for hygienic behavior using proteomic biomarker analysis [17]. Bees exhibiting hygienic behavior are better at removing dead, disease, and/or dying brood, which makes them more resistant to some diseases, including AFB [79]. Guarna et al. not only identified biomarkers for hygienic behavior but employed them to select for a disease‐resistant population. The researchers demonstrated that selectively bred colonies exposed to AFB and *Varroa* mites had a significantly higher survival rate than the control group, making this the first evidence that protein/peptide expression markers can be feasibly utilized for selective breeding, thereby presenting a new direction for proteomic applications.

### 5.2 Fungal diseases

#### 5.2.1 *Vairimorpha* spp

*Vairimorpha* spp., an obligate intracellular parasite from the microsporidia group, poses a threat to *A. mellifera* by impacting individual bees and entire colonies (Figure 3B), altering their physiology, behavior, and survival [61, 80]. Historically, *V. apis* was the only species in this genus to infect *A. mellifera*, but a second species, *V. ceranae*, which normally infected the closely related Eastern honey bee (*A. cerana*) was detected in *A. mellifera* colonies in the early 2000s [81]. The initial case of *A. mellifera* infected with *V. ceranae* was reported in 2006, and it has since become the dominant microsporidian pathogen affecting *A. mellifera* globally [82, 83, 84, 85]. There is a clear association between colony collapse and *V. ceranae*, with risk of hive depopulation being six times higher in infected colonies compared to those uninfected [85, 86]. While *Vairimorpha*‐tolerant and *Vairimorpha*‐sensitive honey bees display similar spore titers, the survival rate of the tolerant lineage was significantly higher than the sensitive lineage, suggesting that tolerance occurs at the individual level, rather than the colony [87, 88, 89].

Three notable *V. ceranae* proteins have been identified in infection comparisons of *Vairimorpha*‐sensitive and tolerant honey bees: hypothetical protein NCER_101994 was found in both *Vairimorpha*‐sensitive and tolerant bees after pathogen challenge, and hypothetical protein NCER_100570 and *Vairimorpha* HSP70 were found in sensitive bees [89]. In addition to these proteins, Kurze et al. also identified an increase in honey bee cytochrome C oxidase subunit 6A1, alpha‐glucosidase precursor, and ATP synthase subunit beta in infected individuals, all of which are important for host energy metabolism. Furthermore, there was a higher abundance of host antioxidants (thioredoxin peroxidase and peroxiredoxin‐like protein) and apoptosis related proteins [84]. NCER_100570 was singled out as a possible candidate for future studies as it contains a signal peptide which may be involved in a crucial infection pathway [89].

Interestingly, despite many studies focusing on *Vairimorpha*’s role in colony loss, its true effect has been called into question in a recent paper [90]. Multivariate analysis revealed a correlation between *Vairimorpha* infection and colony loss; however, its impact is of minimal biological significance when compared to the predominant cause of colony loss, *Varroa destructor* [90]. However, other sources refer to the disease as the “silent killer,” due to its sometimes‐symptomless onset, with poor outcomes [91]. Though the magnitude of its impact is questionable, *Vairimorpha* is still harmful, and understanding the proteins involved in host–pathogen interactions may, in the long run, lead to developing new therapeutics that disrupt *Vairimorpha's* protein machinery or prevent its proteins from interacting with the host [62]. As this pathogen often goes undetected and has a registered treatment (Fumagillin‐B), this is one case where a proactive monitoring strategy (either via microscopy or as part of a multiplexed suite of protein markers) could have a real impact on colony outcomes.

#### 5.2.2 Chalkbrood (*Ascosphaera apis*)

Chalkbrood (Figure 3C) is a fungal disease, caused by *A. apis*, which affects honey bee larvae and can cause serious economic damage, either directly or through interactions with other pathogens [92]. Surprisingly few studies have investigated chalkbrood using proteomics. Though not strictly proteomics, Li et al. conducted a study on the effects of *A. apis* infection on the antioxidant enzyme activities and gut metabolic profiles of honey bee larvae, using ELISA and untargeted metabolomic methods [93]. The study found that the activities of certain antioxidant enzymes, specifically superoxide dismutase, catalase, and glutathione S‐transferase, were significantly greater in the guts of uninfected control larvae compared to those infected with *A. apis*, indicating that infection may impair the infected honey bee larvae's capacity to cope with oxidative stress. The scarcity of proteomic studies on chalkbrood highlights a significant knowledge gap, pointing to the need for further research to elucidate the infection mechanisms associated with this disease.

### 5.3 Virus diseases

#### 5.3.1 Black queen cell virus

Black queen cell virus (BQCV), a prevalent member of the *Dicistroviridae* family, is a poorly understood pathogen that infects honey bee queen larvae and pupae, often causing mortality [94, 95, 96, 97]. The virus can also infect workers and drones but does not cause the characteristic darkened cells of its namesake. Despite its widespread occurrence, BQCV usually remains a latent infection, with symptoms often going undetected until a decline in colony vigor or issues in queen rearing emerge [98]. Symptom‐based diagnosis, while cost‐effective, means that when a diagnosis is eventually made, it is typically too late for remedial action [99] (if a treatment were to exist). This demonstrates the need for rapid molecular testing for early detection of infection before any visible clinical signs are present. Milićević et al. [100] suggest using RT‐PCR to test for the presence of BQCV in honey, which offers simplicity and cost‐effectiveness. Proteomics, especially if already being conducted on a colony for other purposes, may also provide early detection in future health monitoring, along with more comprehensive insights into the colony's overall health.

#### 5.3.2 Sacbrood virus

Sacbrood virus (SBV) is a nonenveloped virus with a capsid consisting of four major proteins VP1, VP2, VP3, and functional analogs of VP4 subunits, as well as a unique minor capsid protein [101]. Much of the research on SBV has focused on genome sequencing, and information regarding the function of SBV proteins and how they affect the host is limited (Figure 3D). Studies have shown that VP2 and VP3 proteins exhibit higher immunogenicity compared to VP1 [102], and VP3 may affect the cleavage of double‐stranded RNA (dsRNA) by inhibiting dicer enzyme activity, thereby playing a role in host RNA interference (RNAi) inhibition [103]. The VP1 protein has been found to interact with heat shock protein 70 cognate 5 (Hsp70‐c5) and may influence SBV infection [104]. Like other viruses, SBV infection triggers rapid increases in the expression of AMPs such as apidaecin, hymenoptaecin, abaecin, and defensin, which are regulated by Toll and Imd/JNK pathways [105]. Upregulation of dicer‐like, argonaute‐2, and bee antiviral protein‐1 have also been observed in response to SBV infection [106, 107]. Incorporating proteomics approaches for identifying and tracking the proteins in question could aid in the development of preventative measures for rapid response to SBV outbreaks, which typically begin asymptomatically and currently have no proven treatment.

#### 5.3.3 Chronic bee paralysis virus

Chronic bee paralysis virus (CBPV), a yet‐to‐be‐fully classified single‐stranded RNA virus, is a viral ailment in adult bees that triggers occult paralysis with discernible behavioral and physiological changes [108, 109, 110]. The difficulty in detecting and intervening in this infection arises from the minimal symptoms presented during its early stages, making it challenging to identify the disease before it progresses to a fatal stage without proactive monitoring. The virus is capable of infecting numerous individuals within a colony, with some showing symptoms days after initial infection and others remaining asymptomatic, allowing CBPV to proliferate undetected. Despite the utilization of RT‐PCR, diagnostic methods often yield inaccurate results and are challenging to replicate consistently [111]. Proteomic research on CBPV is scarce, apart from one study, where the aim was to characterize the structural proteins of CBPV using MS [112]. Given the challenges of diagnosing the disease based solely on phenotype, regular proteomic health monitoring regiments could offer early signs of disease, and deeper insight into the molecular‐level physiology that cannot be fully elucidated through genomics and transcriptomics alone.

#### 5.3.4 Acute bee paralysis virus, Kashmir bee virus, and Israeli acute paralysis virus

Acute bee paralysis virus (ABPV), Kashmir bee virus (KBV), and Israeli acute paralysis virus (IAPV) form a closely related virus group characterized by similar transmission routes, primary host life stages, and a generally low, subclinical presence that often persists undetected within colonies, typical of most dicistroviruses, without clear symptoms at the individual or colony level [113]. However, they are extremely virulent when injected into pupae or adults [114, 115]. Specifically, ABPV and IAPV infections lead to rapid paralysis, tremors, flight incapacity, and a progressive darkening and hair loss on the thorax and abdomen before death, a pattern not observed with KBV [116, 117]. While these viruses can infect larvae and pupae, particularly in colonies with lethal infection levels, with ABPV most frequently found in the brood, they are naturally more common in adult bees [118, 119, 120, 121]. ABPV, KBV, and IAPV are readily identified by ELISA and PCR techniques [122, 123, 124].

Despite the prevalence of these viruses, there is a lack of comprehensive proteomic investigations to supplement and enhance current understanding. A study by Michaud et al. [125] utilized tandem MS on IAPV‐infected bees and found that proteins involved in translation and the ubiquitin–proteasome pathway were most highly enriched. Another study found expression of histone proteins, proteolysis proteins 26‐29‐p, and proteasomal subunits Tbp‐1, Rpn11, Prosα5, and Pros26.4 were altered during infection [126]. As for the other pathogens discussed in this review, studying numerous potential biomarkers through proteomics can enhance our understanding of underlying mechanisms, thereby facilitating the development and implementation of preventative strategies, and enabling early detection via standardized health monitoring regimens. The ability of proteomics to assess many molecular systems at once could also aid in understanding colony wide responses to infection and (eventual) treatment alike, assessing the processes that may occur during the lag time before the colony is healthy again.

#### 5.3.5 Deformed wing virus

Wing deformities in *A. mellifera* (Figure 3E), which are symptomatic indicators of colony collapse, are frequently caused by deformed wing virus (DWV), a single‐stranded RNA virus spread by *V. destructor*. DWV is the principal virus linked to colony dwindling due to unchecked mite infestation [127, 128]. Interestingly, in the absence of *V. destructor*, DWV typically persists at basal levels within the bee colony without obvious detrimental effects, despite still being detected in all life stages, ranging from eggs to adult bees, as well as in glandular secretions used for feeding [129, 130]. Symptoms of DWV infection can extend beyond wing irregularities to include other damaged anatomy, such as truncated and rounded abdomens, discoloration, and paralysis of both the legs and wings [131]. Bees with symptomatic DWV infections typically survive less than 48 h and are commonly expelled from the hive, a phenomenon that correlates with high DWV levels [132].

Promising initial proteomic efforts for monitoring bee health include a study by Di Prisco et al. that found that DWV, transmitted by *V. destructor*, disrupts NF‐κB signaling [133], and two groups that noted that DWV infection downregulated apoptosis‐related genes, suggesting a mechanism for the propagation of the virus throughout the colony [134, 135]. These investigations showcase the ability of proteomics to uncover and assesses *A. mellifera* physiology on a molecular scale.

### 5.4 Parasites

#### 5.4.1 *Varroa* mite

*V. destructor* (commonly referred to as *Varroa*) (Figure 3F) is one of the most pertinent and well‐studied challenges facing honey bees and beekeepers alike; however, there are still large gaps in our understanding of the mite's fundamental biology. To aid in the continued research of the *Varroa* life cycle, an interactive protein atlas was developed by McAfee et al. [136] that encompassed 1433 differentially expressed proteins through the developmental stages of the mites (https://varroa.msl.ubc.ca/index.html).

Recent studies have concentrated on the proteomic impacts of *V. destructor*, examining the differential protein expression patterns in infected workers and drones, as well as proteomic alterations linked to social immunity behaviors [137, 138, 139, 140]. Exposure to *Varroa* mites can effect honey bees early in life, as the larvae exhibit an increased abundance of proteins linked to immunity and stress response upon exposure [141]. Kunc et al. [142] utilized multiomics methods to study the impact of *Varroa* parasitism in 10‐day‐old worker bees, showing transcriptome and proteome changes affecting immunity, oxidative stress, olfactory recognition, sphingolipid metabolism, and RNA regulation; the immune response and sphingolipid metabolism were strongly activated, while olfactory recognition and oxidative stress pathways were inhibited [142]. Another study looking at young bees following *Varroa* parasitism, identified protein changes in the hemolymph, implicating carbohydrate metabolism, detoxification and oxidative stress response, nutrient reservoir activity, oxidoreductase activity, and the olfactory system – suggesting *Varroa* may disrupt many essential pathways from the beginning of life [143].

Surlis et al. [138] utilized label free quantitative proteomics to investigate whether *Varroa* causes differing effects on different castes, finding 202 and 250 differentially abundant in parasitized drone and worker pupae, respectively. Both groups had reduced levels of proteins related to the cuticle, lipid transport, and innate immunity, while metabolic proteins were more abundant, especially in workers [138]. Drones had higher levels of cytoskeletal and muscle proteins, while workers showed increased proteins for fatty acid and carbohydrate metabolism, and ribosomal proteins, indicating different responses or *Varroa* strategies in different castes [138]. Investigating the metabolic and signaling pathways within *Varroa* parasitized bees showing signs of DWV, Erban et al. [144] further showcased the widespread effect of *Varroa*. They revealed that *Varroa* parasitism combined with DWV signs caused proteome changes like *Varroa* alone, but at a greater intensity [144]. They further investigated these proteome changes and found that *Varroa* activates TGF‐β‐induced pathways, suppressing wound healing and immune responses [144].

Studies looking into how the social behaviors of honey bees interact with *Varroa* infestation have led to notable research showcasing proteomics as a tool for selective breeding. Hu et al. [145] provided a thorough examination of *Varroa*‐sensitive hygiene (VSH), a social immunity behavior that protects honey bees against *Varroa* infestation, identifying significant proteomic differences between VSH and non‐VSH honey bees, particularly an enhanced expression of proteins associated with immunity and stress in VSH bees. Furthermore, Parker et al. investigated proteome‐wide changes associated *Varroa* resistance adaptations, showcasing the presence of proteins that predict immunity, both innate and social [137]. Guarna et al. demonstrated the use of selecting for *Varroa* resistance via a protein biomarker panel (composed of 13 proteins with significant associations with hygienic behavior, grooming, and VSH), that resulted in increased resistance to *Varroa* after three generations of selection [17]. This was the first study that was able to successively use protein markers for selective breeding in agricultural organisms and promises a new avenue for selective breeding in not just honey bees, but other animals and plants alike. These studies highlight that proteomics for selective breeding is feasible, and identifying specific predictive markers also deepens our understanding of honey bees intricate social immunity behaviors.

#### 5.4.2 *Tropilaelaps* mites

The *Tropilaelaps* genus consists of four parasitic mite species: *T. clareae*, *T. mercedesae*, *T. koenigerum*, and *T. thaii*, with only the first two infecting *A. mellifera*. While all these mites originate in Asia, *T. mercedesae* is the most worrisome for potentially dispersing to other continents through shipping routes and the global bee trade [146]. Alarmingly, a population of *T. mercedesae* has now been detected in Europe, which dramatically increases the likelihood of dispersal to the Americas through importations [147]. Like *Varroa*, *Tropilaelaps* reproduces in the brood cells; however, due its shorter lifecycle, *Tropilaelaps* can outcompete *Varroa* in warmer climates [146]. As the threat of *T. mercedasae* mites persists, more research will provide crucial foundational knowledge of the mite's biology, and how it compares to existing knowledge of *Varroa*. This kind of information will help determine if miticides or novel RNAi therapies [148] which are being developed to combat *Varroa* will also be effective against *Tropilaelaps*.

#### 5.4.3 Honey bee tracheal mite

The honey bee tracheal mite (*Acarapis woodi*), an obligate endoparasite, contributes to diminished honey production, smaller brood areas, and reduced bee populations, often resulting in colony losses, particularly during winter [149]. While their prevalence in Canada has been low [150], the worldwide prevalence of *A. woodi* has been difficult to ascertain, as this parasite has been overshadowed by *V. destructor* (and perhaps killed from *Varroa* treatments); however, recent studies have noted a likely underestimation of its prevalence in Japan and Italy, possibly alluding to a similar pattern worldwide [151, 152]. While detection methods like RT‐PCR and ELISA exist, there is a scarcity of research on effects of this parasite, especially proteomics based, presenting an opportunity to enhance our understanding of this condition [153, 154].

#### 5.4.4 *Lotmaria passim* trypanosome

Trypanosomatid infections were traditionally attributed to *Crithidia mellificae*; however, recent studies have identified another species, *Lotmaria passim* [155, 156]. While limited information is available on the pathogenicity of *L. passim*, it has been shown that honey bees infected by this parasite have reduced lifespans and it is the most prevalent trypanosomatid parasite in honey bees [157, 158, 159, 160, 161, 162]. Label‐free proteomics studies have discovered that imidacloprid exacerbates *L. passim* infections, increasing both prevalence and parasite load in honey bees [163]. Additionally, the exoproteome (secretome) of *L. passim*, particularly the protein aspartyl protease, has been linked to the infection process in honey bees [164]. Research also indicates that co‐infection with *L. passim* and *N. ceranae* can severely impact bee health, leading to higher mortality rates due to a synergistic effect [165, 166].

## 6 CONCLUSION

The escalating health problems in honey bees, which are vital for global food production and agriculture, underscore the need to unravel cellular mechanisms of pathogenesis and their response to a changing environment. Proteomic studies hold promise for the ongoing surveillance of honey bee wellbeing, offering insights into the various stressors that beehives endure due to climate volatility and the increased prevalence of extreme weather events. The proteomic landscape of how pathogens and their interactions affect *A. mellifera* remains relatively uncharted, despite multipathogen exposure being the norm [167], leaving a substantial lacuna in the existing literature. Bridging these knowledge gaps is not only valuable for identifying novel therapeutic targets but also for reorienting research priorities. There is a compelling case to transition from pathogen or pesticide‐specific investigations toward the identification of broader biomarkers of hive health. Given that honey bees are often subjected to an array of concurrent stressors, proteomics offers an integrative approach to gauge hive health, enabling simultaneous measurement of responses to various stimuli. We posit that proteomics could serve as a comprehensive tool for hive condition appraisal, while also facilitating the detection of diseases through the identification of pathogen‐specific proteins.

## CONFLICT OF INTEREST STATEMENT

The authors have declared no conflict of interest.

## Data Availability

No new data generated here.
